# Haploid Induction in Tomato (*Solanum lycopersicum* L.) via Gynogenesis

**DOI:** 10.3390/plants11121595

**Published:** 2022-06-17

**Authors:** Ivan Maryn Marin-Montes, Juan Enrique Rodríguez-Pérez, Alejandrina Robledo-Paz, Eulogio de la Cruz-Torres, Aureliano Peña-Lomelí, Jaime Sahagún-Castellanos

**Affiliations:** 1 Departamento de Fitotecnia, Universidad Autónoma Chapingo, Chapingo 56230, Mexico; maryn.montes@gmail.com (I.M.M.-M.); erodriguezx@gmail.com (J.E.R.-P.); penalomeli@gmail.com (A.P.-L.); 2 Posgrado en Recursos Genéticos y Productividad, Colegio de Postgraduados, Montecillo 56230, Mexico; arobledo@colpos.mx; 3 Departamento de Biología, Instituto Nacional de Investigaciones Nucleares, Ocoyoacac 52750, Mexico; eulogio.delacruz@inin.gob.mx

**Keywords:** doubled haploids, in vitro ovule culture, irradiated pollen, wide hybridization, in vivo haploid inducers, breeding

## Abstract

The generation of new hybrid varieties of tomato (*Solanum lycopersicum* L.) is the most widely used breeding method for this species and requires at least seven self-fertilization cycles to generate stable parent lines. The development of doubled haploids aims at obtaining completely homozygous lines in a single generation, although, to date, routine commercial application has not been possible in this species. In contrast, obtaining doubled haploid lines via gynogenesis has been successfully implemented in recalcitrant crops such as melon, cucumber, pumpkin, loquat and walnut. This review provides an overview of the requirements and advantages of gynogenesis as an inducer of haploidy in different agricultural crops, with the purpose of assessing the potential for its application in tomato breeding. Successful cases of gynogenesis variants involving in vitro culture of unfertilized ovules, use of ^60^Co-irradiated pollen, in vivo haploid inducers and wide hybridization are presented, suggesting that these methodologies could be implemented in tomato breeding programs to obtain doubled haploids.

## 1. Introduction

Tomato (*Solanum lycopersicum* L.) is one of the most consumed vegetable species worldwide, which is reflected in its production in 2019 when just over five million hectares were planted, yielding 180,166,329 tons of fruits [1]. This wide diffusion in production areas with the presence of various adverse factors continuously demands improved varieties that provide the possibility of increasing the production and quality of the fruit, with the necessary characteristics to carry out the appropriate agronomic management. This is a constant task within the world seed market in which the plant breeder is obliged to design strategies to reduce the time required to obtain new commercial varieties.

The vegetable seed market is increasingly offering hybrid varieties that allow obtaining better yields and fruit quality by taking advantage of the phenomenon of heterosis and by immediately combining characteristics of both parents. This methodology, in its classic version, requires obtaining pure lines, or populations with high inbreeding, to ensure the stability of the generated genotype [2,3]. The classical breeding of autogamous species, such as tomato, requires the execution of several stages: an initial cross to generate genetic variability, the selection of segregating genotypes to obtain homozygous lines with traits of agronomic interest, the identification of combinations between parent lines with a high expression of heterosis, the evaluation of yield and quality and stability of the hybrids under production conditions and the final release of the improved hybrid variety.

In this process, obtaining lines alone requires at least seven self-fertilization cycles [3,4], which means that the time invested to release a new variety occurs in a period of 11 to 13 years [3]. This makes it necessary to develop alternatives to reduce the time to obtain homozygous genotypes. Currently, two main techniques are used: (a) doubled haploids (DHs) and (b) the fast generation cycle system (FGCS) [3]. Of these options, doubled haploids are the most widely used in different agricultural crops due to their efficiency in obtaining pure lines in crops such as *Oryza sativa* L. [5,6], *Zea mays* L. [7,8,9] and *Triticum aestivum* L. [10,11].

The generation of doubled haploids through androgenesis has not had the expected results in some vegetables of high economic value such as chili pepper (*Capsicum* spp.) [12], eggplant (*Solanum melongena* L.) [13], tomato [2,4] and cucurbits [14]. In tomatoes, studies have been carried out to induce haploidy by another culture, although satisfactory results have not been obtained to allow its routine application in breeding programs [4,15]. However, given the possibility of substantially reducing the time to obtain lines by using this technique, it is attractive to continue the search for alternatives that allow for efficiently obtaining DHs in this crop. 

Gynogenesis is a viable methodology with promising results in recalcitrant species for the generation of doubled haploids, which uses unpollinated female gametophytes [14,16]. This technique has been successful in loquat (*Eriobotrya japonica* (Thumb) Lindl.) [17], citrus (*Citrus grandis* (L.) Osbeck) [18], spinach (*Spinacia oleracea* L.) [19], cucurbits [14,20], red beet (*Beta vulgaris* L.) [21] and *Gentiana* ssp. [22] crops, where it is feasible to apply this technique in breeding.

However, in tomatoes, results that support the routine use of gynogenesis have not yet been found. Therefore, this literature review focuses on the advantages of gynogenesis, its requirements, success stories and the state of the art in the generation of doubled haploid plants in tomatoes.

## 2. Importance of Doubled Haploids

Doubled haploid (DH) produces homozygous genotypes in a single generation. The idea is to generate a haploid genotype with a single set of *n* chromosomes; subsequently, through chromosomal duplication, genotypes with *2n* chromosomes are generated. These plants have two identical chromosome sets and, consequently, are homozygous at each of their loci [23,24]. To achieve the above, the protocols reported worldwide coincide in the following procedure: (a) induction of haploidy by in vitro culture of anthers [5], ovules [12,13] or in vivo induction [9,25]; (b) duplication of chromosomal material by colchicine [26]; and (c) determination of haploidy level by squash root apices and/or flow cytometry [5,7,17].

The generation of homozygous individuals by haploid doubling has become a useful and efficient tool in the breeding of plants such as maize [27,28], wheat [29,30] and rice [31,32]. The main reasons for using DHs in breeding programs are: (1) obtaining pure lines in a single generation, (2) fixing desirable genotypic combinations, (3) increasing selection efficiency, and (4) reducing sample size for selection. These advantages help reduce the time and cost of breeding, which can represent savings in the order of millions of dollars, even in the case of small breeding programs, by substantially increasing the achievement of outstanding results [23]. 

Several in vitro tissue culture techniques have been developed in different species of agricultural interest for the generation of doubled haploids for commercial purposes. Of these, androgenesis is the most reported worldwide [23,24]. However, the results of this methodology are varied due to the little or no response of anthers to in vitro culture conditions (recalcitrance). A recalcitrant species is one in which morphogenetic processes such as somatic embryogenesis or organogenesis are not successful, and therefore, it is not possible to regenerate plants even when provided with favorable culture conditions [33]. For this reason, recalcitrance is the main problem for haploidy induction by in vitro anther culture. Among the species of agricultural interest with this problem are some *Solanaceae* [2] and *Cucurbitaceae* [14]; therefore, the search for alternatives to induce haploids continues to reduce breeding processes for the development of new commercial varieties.

## 3. Doubled Haploids in Tomato

Obtaining DHs in tomato has been the subject of research for more than 30 years due to the economic importance of the crop; however, no standardized, efficient and reproducible protocols for generating doubled haploids in this vegetable have been reported in the literature reviewed. Among the factors identified that prevent the achievement of this objective are recalcitrance to in vitro culture [4,15] and polyploidy generated by the fusion of nuclei [34,35]. It is still necessary to define the incubation conditions, the physical and chemical conditions of the medium, the genotype dependency of the in vitro culture, the physiological state of the mother plant, and anther development, which affect the repeatability of protocols to achieve the induction of haploid plants [2,15]. The first published studies on androgenesis in tomato reported that it is possible to obtain haploid callus and maintain it for 7 months, although with the subsequent morphogenetic induction of roots only [36]. Subsequently, it was reported that it is feasible to obtain haploid plants by in vitro culture of anthers of *S. pimpinelilfolium* L. and *S. peruvianum* L. [37]. Their success was in choosing the correct developmental stage of the anthers, which is between metaphase I and telophase II of microspore development [38].

Different evaluations of the in vitro culture of tomato anthers led to the conclusion that it is possible to obtain plants by organogenesis (adventitious shoots) and that this would allow accelerating their breeding, although it is still not an efficient way for haploid induction due to the few replicable results [39,40]. The generation of haploids in tomato is not efficient because anthers generate three types of callus (an irregular mass of parenchyma tissue that, despite lacking structure, allows cell differentiation), which was induced by 4.4 g·L^−1^ of Murashige and Skoog medium, 20 g L^−1^ sucrose, 1 mg·L^−1^ 2-isopenteniladenina and 2 mg·L^−1^ indoleacetic acid, where the calli that presented polygonal cells with a large vacuole and faster growth became amorphous and friable macroscopic masses with a rough and granular surface where only 7% of cells were haploid [41]. Similarly, it was shown that from gametophytes and sporophytes it is possible to obtain calli; however, in these the nuclei fuse, leading to polyploidy [42]. Likewise, Corral-Martínez et al. [34] obtained calli by culturing 4- to 5-mm-long anthers of the ms1035 mutant in an induction medium composed of 4.4 g·L^−1^ of Murashige and Skoog medium, 2.5 g·L^−1^ phytagel, 20 g·L^−1^ sucrose, 1 mg·L^−1^ 2-isopentenyladenine and 2 mg·L^−1^ indoleacetic acid; however, they did not obtain a favorable response, and only one plant was haploid out of the 83 regenerated, which was attributed to the fact that the growth rate of callus of somatic origin is higher compared to those generated from meiocytes.

Based on these advances, further research has been carried out to improve protocols for generating doubled haploids. For example, Moreno et al. [43] determined that in tomato it is possible to culture anthers in vitro with microspores from the tetrad stage to the uninucleate stage by using the regulators 2-isopentenyladenine (1 mg·L^−1^) and indoleacetic acid (4 mg·L^−1^); with this, the *2n* androgenic plants generated was 97%, although the fusion of the meiocyte nuclei generated the loss of complete homozygosity of the plants obtained. Thus, Niazian et al. [15], using the artificial neural network model for image processing, found that the main factors determining androgenesis and callus induction and regeneration are the genotype and the concentration of 2,4-dichlorophenoxyacetic.

Due to the recalcitrance of tomato, the results achieved in androgenesis so far have not been favorable or replicable; therefore, there is still no reliable and efficient methodology for haploid induction. However, due to the great advantages that DHs represent in breeding, it is necessary to continue searching for successful alternatives for this purpose (Table 1).

## 4. Gynogenesis in Agricultural Crops

Haploid regeneration by means of unpollinated female gametophytes is one of the most commonly used alternatives in species where androgenesis has not been effective; this method is called haploid gynogenesis or haploid parthenogenesis. The term gynogenic haploid regeneration is used for all haploid induction methods in which a female gametophyte is used as the origin of the haploid cells, regardless of whether it is a pseudofertilization process or not; therefore, there are four variants: (a) in vitro culture of unfertilized ovaries or ovules [14], (b) pollination with pollen irradiated with cobalt-60 (^60^Co) [16,20], (c) wide hybridization [16] and (d) in vivo haploid inducers [20,25].

### 4.1. In Vitro Culture of Ovaries or Ovules

In the case of self-pollinated species, in vitro culture of unfertilized female gametes is achieved by culturing flower buds prior to anthesis, while in male-sterile or self-incompatible plants it is performed at any stage of ovule development, since they show a favorable response to gynogenic induction [24]. This technique is successfully employed in species of the genus *Allium*, where it is the main technique to derive DHs [44]. For example, Panahandeh et al. [45] achieved a gynogenic induction range of 5 to 12% by culturing unpollinated flower buds of *Allium hirtifolium* Boiss., which allowed callus formation with a success rate of 20%, of which the efficiency of obtaining haploid plants was 70 to 77%. This technique is also viable in both wild and improved species of the genus *Gentiana* L. spp. [22,46,47]. Although the results obtained were promising in both species mentioned, the authors agree that it is necessary to continue with the establishment of efficient protocols because the average response in obtaining haploid plants does not exceed 5% (Table 2).

### 4.2. Irradiated Pollen

Irradiated pollen allows the development of haploid embryos by fertilizing an ovule with mature pollen whose genetic material is inactive, i.e., it is capable of inducing cell divisions in the ovule and the normal development of the embryo [16]. There are many favorable examples involving the use of irradiated pollen in different vegetable and fruit species in which androgenesis was not an option (Table 3).

Thus, Hooghvorst et al. [20] and Kurtar et al. [53] reported that in cucurbits, a family containing crops of high economic value such as pumpkin, melon and cucumber, pollination with γ-ray-irradiated pollen is the most efficient method to induce haploidy because it has not been possible to take advantage of androgenesis in these crops. In *Cucumis melo* L., pollen irradiated with 250 Gys of ^137^Cs was more effective compared to in vitro culture of unpollinated ovules [50]. Likewise, Nasertorabi et al. [51] obtained 48 Cucumis melo L. plants induced from embryos obtained with pollen irradiated with 550 Gys of ^60^Co, of which 94% were haploid.

In citrus, this technique has proven to be very useful to obtain haploid plants with high value for breeding. For example, Wang et al. [49] were able to induce haploid plants in *Citrus grandis* L. *Osbeck* by irradiating pollen with γ-rays with doses lower than 500 Gys and in vitro culture of immature embryos. Likewise, Jedidi et al. [52], by irradiating pollen at 250 Gys with γ-rays, obtained seven seedlings that were used to generate homozygous lines in *Citrus reticulata* Blanco.

### 4.3. Wide Hybridization

The third variant of gynogenesis consists of interspecific crosses, through which it is possible to induce the formation of haploid embryos due to the fertilization of an ovule with pollen from a distant species, allowing double fertilization. However, cell divisions in the zygote eliminate the chromosomes of the male parent [16,54]. Thus, Santra et al. [55] published an efficient protocol to obtain completely homozygous lines in only two years by wide hybridization to obtain DHs from wheat pollinated with maize pollen. 

Although wide hybridization is most commonly used in cereals, in recent years its application in leafy vegetables has been shown to have acceptable results in the induction of haploid plants (Table 4). For example, Piosik et al. [56] carried out distant hybridization of *Lactuca sativa* L. with *Helianthus annus* L. and *Helianthus tuberosus* L., with which they established an effective methodology to induce haploidy in lettuce. In addition, Wei et al. [57] obtained haploid offspring by embryo rescue and subsequent duplication of chromosomal material with colchicine using a commercial variety of *Brassica oleracea* var. *alboglabra* as the male parent and a variety of *Brassica rapa* var. *parachinensis* as the female parent. Similarly, haploid plants were obtained by crossing *Brassica rapa × Brassica oleracea* and in vitro culture of immature embryos [58].

### 4.4. In Vivo Haploid Induction

In the past decade, methodologies applied to induce in vivo haploidy to accelerate the production of double haploid lines have been developed for several target crops [20,61]. These methodologies take advantage of the specific gene expressions that regulate the formation of maternal haploids (Table 5). In maize, the generation of in vivo haploid inducer lines of maternal haploidy via the expression of the genes *MATL* [9], *NLD* [62] and *ZmPLA1* has been possible [63]. In wheat, the genetic edition of the gen *MTL* permitted to observe that the alleles *mtl-AD*, *mtl-BD* and *mtl-ABD* were effective to generate inducer lines from self-pollinated and cross-pollinated progenies; its rate of success ranged between 7.8% and 15.6% [64]. However, these genes do not work in dicot species [65]. On the other hand, the haploid induction from aneuploidy is possible via CRISPR/Cas9 mutation of the *CENH3* gene in both monocot and dicot crops [20,61]. These two methodologies are very promising and are used in cereals because they have been more efficient than the in vitro methods.

In contrast, the conservation of the *DMP* genes in dicot species opens up the possibility to apply this haploidy induction system [65]. From this starting point, protocols for some horticultural crops have been developed. In *Brassica napus* L., *bnaDMP* mutation could induce amphihaploidy [66,67]. In *Nicotiana tabacum* L., it was reported that the simultaneous *MtDMP1*, *MtDMP2* and *MtDMP3* mutations can trigger maternal haploidy at rates from 1.52% to 1.75% [68]. In contrast, the inactivation of the *MtDMP8* and *MtDMP9* alleles in *Medicago truncatula* Gaertn would facilitate in vivo maternal haploid induction at a rate from 0.29% to 0.82% in mutant progeny [69]. Despite these results, the use of *DMP* genes is not very frequent because there are not transformation systems (CRISPR/Cas9) or TILLING populations in major crops [20,67]. 

## 5. Gynogenesis in Tomato

Due to the few successful results obtained by androgenesis for haploidy induction and the formation of doubled haploids in tomato, some research groups have sought alternatives to achieve this goal. The options employed are variants of gynogenesis: wide hybridization, unfertilized ovule culture and irradiated pollen [4]; and haploid inducers/CRISPR/Cas9 [70]; however, it is not yet fully known what these could mean for the breeding of this crop.

### 5.1. Wide Hybridization

Wild species phylogenetically related to tomato are commonly used for crop improvement to incorporate alleles of interest into crop breeding programs, most notably *S. pimpinellifolium* [72], *S. arcanum* Peralta [73], *S. sitiens* I. M. Johnst [74], *S. pinnelli* L. [75], *S. chilense* (Dunal) Reiche [76], *S. neorickii* D. M. Spooner, G. J. Anderson & R. K. Jansen [77], *S. habrochaites* S. Knapp & D. M. Spooner [78] and *S. sisymbriifolium* Lam. [79].

The general use of wide crosses in this species is not only performed to induce haploidy, as some studies have attempted to apply them to generate DHs (Table 4). For example, *S. sisybriifolium* pollen was used unsuccessfully to induce haploids [59]. In contrast, *S. sisybriifolium* pollen allowed obtaining haploid and di-haploid genotypes of maternal origin.

Even though only ~10% of embryos were rescued and only two plants were generated, the results suggest that it may be a viable alternative; however, the author suggests that the procedure needs to be modified to improve results [60].

### 5.2. Unfertilized Ovule Culture

Few attempts have been made to obtain haploid tomato plants by in vitro culture of unfertilized ovules (Table 2). In tomato, this objective was not possible despite the fact that ovules have a variable response to different culture media [48]. Moreover, Zhao et al. [80] designed a very efficient in vitro protocol with which they isolated, from a single ovary in tomato, between 100 and 150 ovules with which they were able to induce gynogenic callus; despite this, they were unsuccessful in regenerating haploid plants.

### 5.3. Irradiated Pollen

Regarding the use of irradiated pollen in tomato, the work carried out is limited, although the results are promising (Table 3). Thus, Nishiyama et al. [81] reported that *S. pimpinellifolium* pollen maintains its germination capacity and that it is possible to generate fruits with some seeds with doses of 2000 to 7000 Gys of X-rays. In addition, Nishiyama et al. [82], when applying between 100 and 1100 Gys in increments of 100 Grays with X and γ radiation to *S. pimpinellifolium* pollen, found that it has the same effect on germination and fruit set, with a pollen germination capacity of less than 50% with doses higher than 300 Gys. These studies suggest the possibility of obtaining tomato fruits and seeds from irradiated pollen, although the doses used did not allow inactivating the genetic material of the microspore and inducing haploid parthenogenesis. However, the success of this technique obtained in other crops allows us to assume that it is essential to determine the median lethal dose (LD_50_), which could vary according to the genotype and species [14,18]. 

For this methodology to be used in tomato breeding programs for haploidy induction, the optimum dose for the inactivation of genetic material in pollen must be determined. In recent years, Akbudak et al. [83] irradiated pollen from different tomato hybrids with doses of 100, 200, 300 and 400 Gys of γ-rays without obtaining fruit in any treatment although radiation doses higher than 200 Gys correspond to LD50. Likewise, Bal et al. [4] mentioned their own unpublished work on haploidy induction in this crop using irradiated pollen, where 1000 Gys caused the loss of viability and germination capacity of the pollen; however, with 800 Gys, fruits were generated, which were aborted in the early stages of development.

### 5.4. In Vivo Haploid Inducers

In tomato, the use of CRISPR/Cas9 has been applied to achieve objectives such as introgression breeding [84], plant architecture, fruit development and ripening [85], herbicide-resistance [86], leaf development [87] and ToBRFV-resistant tomato [88]. This suggests that it is possible to generate protocols to use the *DMP* and *CENH3* genes that regulate the gynogenesis to facilitate the generation of maternal haploid inducer males, as reported in maize [9,62,63] and wheat [64]. Thus, Zhong et al. [70] obtained *sldmp* tomato mutants using CRISPR/Cas9, with a rate of 1.9% for haploidy induction. Likewise, KEYGENE N. V. (Wageningen, Netherlands) has a patent for a methodology to generate haploids via GFP-tailswap disruption that by editing the *CENH3* gene produces 0.5–2.3% of haploids [71]. These achievements produced by genetic edition show the potential of the in vivo haploid inducers to obtain DH lines in tomato and other recalcitrant crops.

## 6. Future Perspectives

Once haploid induction is possible in tomato, the next step would be to restore the chromosomal level of the crop to obtain 100% homozygous lines. For this, as is routinely performed in other species, colchicine could be used. It is expected that this would not be an obstacle for the generation of doubled haploids in tomato because the use of colchicine has allowed obtaining tetraploid genotypes [89,90,91]. Therefore, it is necessary for researchers to generate the appropriate methodology for the induction of haploid plants.

Based on the information presented in this paper, it is possible to infer that the use of irradiated pollen may be an option to develop a method to induce haploidy and subsequent development of DH lines in crops recalcitrant to in vitro culture; however, to be used in tomato, it is necessary to determine, through radiosensitivity studies, the appropriate radiation dose for the formation of haploid seeds or embryos. Thus, previous work on this species offers a basis for designing experiments to determine the LD_50_ in pollen; for this, it is suggested to use doses of less than 200 Gys of ^60^Co. Simultaneously, studies should be carried out to determine both the appropriate culture medium that allows in vitro development of immature embryos and the period between pollination and embryo rescue for their culture to generate haploid plants.

The use of the genetic edition with CRISPR/Cas9 in tomato may lead to the development of efficient protocols to activate the *DMP* and *CENH3* genes involved in the gynogenesis regulation. If the new protocols were more successful in inducing haploidy than the existing ones for dicots, their use in plant breeding would facilitate obtaining fully homozygous genotypes in a short time.

## 7. Materials and Methods

The search for scientific information was carried out using the following keywords in English and their equivalents in Spanish: *Solanum lycopersicum* L., tomato, doubled haploid, haploid induction, androgenesis, anther culture, gynogenesis, haploid embryo, embryo rescue, pollination with irradiated pollen, unfertilized ovule, ovary culture, irradiated pollen, pseudofertilized ovule culture, in vivo haploid inducer, maternal haploids, CRISPR/Cas9 haploid and wide hybridization. The search was carried out in different databases: Scopus, Web of Science, SciELO, Springer Link, ScienceDirect, Conricyt, Crossref, EBSCO, PubMed, Taylor and Francis, Wiley Online Library, Google Scholar, Dialnet, Redalyc and ResearchGate. A total of 350 written works were consulted of which 91 were selected, including books, scientific or review articles and scientific notes related to the use of methodologies for the generation of doubled haploids and to success stories in breeding programs for different agricultural crops.

## 8. Conclusions

Doubled haploids are an alternative with great advantages for breeding. Their application in tomato has not yet been possible in a conventional way. A promising technique is to use gynogenesis techniques, specifically irradiated pollen, which together with the rescue of immature embryos and in vitro culture may be an efficient means of achieving this objective. However, novel approaches such as the use of CRISPR/Cas9 mutation system, and its obtained successful results in editing *DMP* and *CENH3* genes trigger in vivo haploidy induction, but it still needs to be explored. Therefore, both methodologies to induce haploidy are to be considered in tomato to achieve its application in breeding programs to take advantage of DHs in terms of time and cost, which would be greatly appreciated by breeders. 

## Figures and Tables

**Table 1:** Reports of androgenesis methodology in *Solanum lycopersicum* L.

Species	Common Name	Pathway	Ploidy Level Determination	Haploid Plants	Reference
*S. lycopersicum* L.	Tomato	Anther culture	Flow cytometry	3	Corral-Martínez et al. [34]
		Anther culture	Flow cytometry	0	Julião et al. [35]
		Anther culture	Burn’s technique	0	Sharp et al. [36]
		Anther culture	Flow cytometry	0	Seguí–Simarro et al. [38]
		Anther culture	Flow cytometry	0	Seguí–Simarro et al. [41]
		Anther culture	Flow cytometry	0	Seguí–Simarro et al. [42]
		Anther culture	Flow cytometry	0	Moreno et al. [43]

**Table 2:** Examples of protocols used for successful haploid induction mediated in vitro culture of unfertilized ovaries or ovules.

Species	Common Name	Pathway	Ploidy Level Determination	Haploid Induction Rate	Reference
*Beta vulgaris* L.	Red beet	Unfertilized ovule culture	Flow cytometry and chromosome counting	25%	Zayachkovskaya et al. [21]
*Gentiana* spp.	Gentians	Unfertilized ovule culture	Flow cytometry and molecular marker analysis	32.5%	Takamura et al. [22]
*Allium hirtifolium* Boiss	Persian shallot	Unfertilized ovary	Squash root	0–77%	Panahandeh et al. [45]
*Gentiana triflora*	Gentians	Unfertilized ovules	Flow cytometry and Feulgen staining	23.5–56%	Doi et al. [47]
*Solanum lycopersicum* L.	Tomato	Non-fertilized ovary culture	-	0%	Bal et al. [48]

**Table 3:** Examples of successful haploid induction methods by induced parthenogenesis by irradiated pollen in recalcitrant species.

Species	Common Name	Pathway	Ploidy Level Determination	Haploid Induction Rate	Reference
*Eriobotrya japonica* (Thunb.) Lindl.	Loquat	γ–irradiated pollen	Flow cytometry	0.007–0.008%	Blasco et al. [17]
*Citrus grandis* (L.) Osbeck	Pummelo	γ–irradiated pollen	Flow cytometry	1%s	Wang et al. [49]
*Spinacia oleracea* L.	Spinach	γ-irradiated pollen	Flow cytometry	-	Keleş et al. [19]
*Cucumis melo* L.	Melon	γ-irradiated pollen	Flow cytometry	14–33%	Lotfi et al. [50]
*Cucumis melo* L.	Melon	γ-irradiated pollen	Chromosome counting	23.65%	Nasertorabi et al. [51]
*Citrus reticulata*	Mandarin	γ-irradiated pollen	Flow cytometry	2.58–8.33%	Jedidi et al. [52]

**Table 4:** Summary of haploid induction methodologies by wide hybridization.

Species	Common Name	Pathway	Ploidy Level Determination	Haploid Induction Rate	Reference
*Triticum aestivum* L.	Wheat	Wheat × maize crossing	-	-	Wiśniewska et al. [10]
*Lactuca sativa* L.	Lettuce	Cross-pollination with *Helianthus annus* L.	Flow cytometry and chromosome counting	15%	Piosik et al. [56]
*Lactuca sativa* L.	Lettuce	Cross-pollination with *Helianthus tuberosus* L.	Flow cytometry and chromosome counting	16%	Piosik et al. [56]
*Solanum lycopersicum* L.	Tomato	Cross-pollination with *S. sisymbriifolium* Lam.	Chromosome counting	0%	Bal et al. [59]
*Solanum lycopersicum* L.	Tomato	Cross-pollination with *S. sisymbriifolium* Lam.	Flow cytometry and chromosome counting	~10% cells haploids	Chambonnet [60]

**Table 5:** Summary of haploid induction reports via in vivo haploid inducers.

Species	Common Name	Pathway	Ploidy Level Determination	Haploid Induction Rate	Reference
*Zea mays* L.	Maize	Inducer inbred lines	Morphological markers	2.5–15.7%	Qu et al. [7]
*Zea mays* L.	Maize	BHI Bulk	Embryo coloration (R1-nj)	11.2–16.8%	Trampe et al. [8]
*Zea mays* L.	Maize	Frame-shift mutation in *MATRILINEAL* (*MTL*)	Flow cytometry	6.7%	Kelliher et al. [9]
*Zea mays* L.	Maize	Eliminate native CENH3- gene	Flow cytometry	0.05–0.31%	Kelliher et al. [25]
*Zea mays* L.	Maize	Inducer lines (NOT LIKE DAD)	Morphological markers	0–3.59%	Gilles et al. [62]
*Triticum aestivum* L.	Wheat	Edited the MTL alleles using CRISPR/Cas9	Chromosome counting	0–15.6%	Tang et al. [64]
*Arabidopsis thaliana*	Arabidopsis	Edited the DMP genes using CRISPR/Cas9	Flow cytometry	0–4.41%	Zhong et al. [65]
*Brassica napus* L.	Oilseed rape	Knocked out of BnaDMP using CRISPR/Cas9	Flow cytometry	1.5 +-0.63%	Li et al. [66]
*Brassica napus* L.	Oilseed rape	DMP CRISPR/Cas9 mutagenesis	Flow cytometry	0–4.44%	Zhong et al. [67]
*Nicotiana tabacum*	Tobacco	DMP CRISPR/Cas9 mutagenesis	Flow cytometry	0–1.63%	Zhong et al. [67]
*Nicotiana tabacum*	Tobacco	DMP CRISPR/Cas9 mutagenesis	Flow cytometry and cytological observation	1.52–1.75%	Zhang et al. [68]
*Medicago truncatula* Gaertn	Barrel medic	DMP CRISPR/Cas9 mutagenesis	Flow cytometry	0.29–0.82%	Wang et al. [69]
*Solanum lycopersicum* L.	Tomato	DMP CRISPR/Cas9 mutagenesis	Flow cytometry	0.5–3.7%	Zhong et al. [70]
*Solanum lycopersicum* L.	Tomato	Edition of the CENH3 gen with GFP-tailswap disruption	Flow cytometry	0.2–2.3%	Op Den Camp et al. [71]

## Data Availability

Not applicable.
